# Balancing individual trade-offs for population gain: optimizing nitrogen and density secures fresh ear yield in rain-fed waxy maize

**DOI:** 10.3389/fpls.2026.1831472

**Published:** 2026-05-22

**Authors:** Chunxia Jiang, Jiangning Yu, Huatao Liu, Na Li, Enke Liu

**Affiliations:** 1 Shanxi Institute of Organic Dryland Farming, Shanxi Agricultural University, Taiyuan, Shanxi, China; 2 Key Laboratory of Sustainable Dryland Agriculture of Shanxi Province, Taiyuan, Shanxi, China; 3 College of Resources and Environment, Shanxi Agricultural University, Jinzhong, Shanxi, China

**Keywords:** planting density, nitrogen application, fresh ear yield, dry matter, nitrogen

## Abstract

**Context:**

Fresh waxy maize is a unique type of maize harvested at the late R3 stage. Both fresh ear yield and weight per ear determine its economic value. Nitrogen (N) application and planting density are the two major agronomic practice factors influencing maize growth and yield; however, optimal values for fresh waxy maize remain poorly defined, particularly in rain-fed areas.

**Objective:**

The purpose of the present study was to clarify the effects of planting density and N application rate on fresh ear yield, in addition to dry matter (DM) and N accumulation and partitioning, and to identify the primary drivers of the effects in fresh waxy maize.

**Methods:**

A two-year field experiment was conducted in Shanxi province, China, using a split-plot design with three replicates. N application rate (0, 120, 180, and 240 kg ha^−1^) was the main factor and planting density (45, 000 and 60, 000 plants ha^−1^) was the second factor.

**Results:**

Increasing planting density from 45, 000 to 60, 000 plants ha^−1^ imposed a trade-off: it suppressed dry matter (DM) and nitrogen (N) accumulation per plant while boosting total DM and N accumulation at population level. However, the optimal N application rate (180 kg ha^−1^) alleviated the negative effects of high planting density (60, 000 plants ha^−1^), ensuring that weight per ear was comparable to that under low density (45, 000 plants ha^−1^). The combination of 180 kg N ha^-1^ and 60, 000 plants ha^-1^ achieved the highest fresh ear yield, demonstrating an effective balance between individual performance and population gain. Therefore, the 180 kg ha^−1^ N and 60, 000 plants ha^−1^ combination is recommended as an efficient management strategy for improving fresh waxy maize productivity and N partial factor productivity.

**Conclusion:**

The findings of the present study provide practical guidance for integrated management of N and planting density in fresh waxy maize to regulate DM, N and yield in the study area and in other regions with similar climatic conditions.

## 1 Introduction

Fresh waxy maize (*Zea mays* L. var. *ceratina* Kulesh) is a unique type of maize harvested in the late R3 stage. China is the origin of waxy maize and the world’s largest producer and consumer of fresh maize, with the planting area for fresh maize expanding considerably over the past decade (Shi et al., 2019). The development of fresh waxy maize is of great significance to the development of the maize production industry and increase in farmer income. Waxy maize yield and quality are impacted not only by cultivar genetic factors but also agronomic practices (Gao et al., 2022; Lou et al., 2024). Among them, nitrogen (N) fertilizer and planting density are the major factors affecting yield (Fan et al., 2023; Liu et al., 2023a; Shi et al., 2016). Furthermore, maize yield formation is driven by dry matter (DM) and nutrient accumulation and allocation (Duan et al., 2023; Shao et al., 2024). Hence, investigating DM and N accumulation and partitioning is crucial to understanding yield formation in fresh waxy maize and achievement of higher productivity under different N fertilizer and planting density conditions.

Increasing planting density is an agronomic practice used to enhance grain yield, as it can increase leaf area index (LAI), in turn increasing radiation interception and high total N uptake for high DM accumulation in the population (Shi et al., 2016; Wu et al., 2024). Furthermore, an optimal planting density can resolve conflict between individual plant growth and population yield by increasing ear number per unit area to compensate for the negative impacts on weight per ear (Li et al., 2025; Yan et al., 2024). However, excessively high planting density intensifies competition for water, nutrients, and light (Zhai et al., 2018). Such competition leads to excessive canopy shading, reducing light penetration to lower leaves, accelerating leaf senescence, impairing photosynthesis, and ultimately limiting DM accumulation and yield (Djaman et al., 2022; Liu et al., 2016). Furthermore, the optimal planting density differs across fields due to differences in soil type, weather, and other management practices (Kalogeropoulos et al., 2024; Zhang et al., 2020). Therefore, determining the optimal planting density for fresh waxy maize is crucial for balancing individual plant development and population yield, particularly in rain-fed areas with limited water resources.

N is a vital and often limiting nutrient for crop growth and development. In maize cultivation, adopting a high planting density is a common strategy for improving production. However, it also intensifies intra-specific competition for resources, frequently leading to reductions of both N acquisition and DM accumulation per plant. Conversely, appropriate N fertilizer application can mitigate such stress, in turn increasing maize DM accumulation, grain yield, LAI significantly (Shi et al., 2016). Although the role of N fertilizer in increasing crop yields is widely recognized, overestimation of the effects of N fertilizer has led to application of high amounts in an effort to achieve high yield (Cui et al., 2018; Ren et al., 2025; Shi et al., 2016; Zhang et al., 2015). Nevertheless, evidence suggests that high yield does not necessarily require a high N rate, because soil N supply can increase under favorable growing conditions (i.e., biological buffering capacity) (Morris et al., 2018). Moreover, excessive N application could result in low N use efficiency, have severe negative impacts on the environment, and impose considerable challenges for sustainable agricultural development (Ai et al., 2024; Yue et al., 2022). Consequently, exploring the appropriate amount of N fertilizer that matches high planting density is imperative for sustainable agriculture.

Previous studies on fresh waxy maize have focused primarily on single factors, such as fertilization (Li et al., 2022a), interaction effects of water deficit and planting density (Zhang et al., 2020), and substitution of organic fertilizer with chemical N fertilizer (Gao et al., 2022; Lou et al., 2024). For fresh waxy maize, the market for individual ears is more akin to vegetable production, where both population yield and individual ear weight determine market value. Therefore, the trade-off between individual plant performance and population productivity becomes particularly critical. High planting density increases ear number per unit area but risks reducing weight per ear. Whether optimal N management can offset this individual-level trade-off to achieve both high population yield and marketable ear weight in rain-fed areas remains unclear. To address the knowledge gap above, a two-year field experiment was carried out in central Shanxi province, which is the main fresh waxy maize planting area in China. The objectives of the present study were to (1) clarify the effects of two planting densities and four N application rates on fresh ear yield, N fertilizer partial productivity (NFPP), and DM and N accumulation and partitioning, and (2) analyze the relationships between yield and DM and N accumulation and partitioning. The findings of the present study could provide a practical guidance for the effective management of fresh waxy maize in rain-fed areas.

## 2 Materials and methods

### 2.1 Experimental location

The field experiment was conducted at Shanxi Organic Dry Farming Research Institute, Shanxi Agricultural University, Taiyuan, China (38°04′ N, 112°89′ E; 1270.0 m above sea level). The region falls under the semiarid-warm temperate continental monsoon climate zone, with an average annual temperature of 6–7 °C. The average annual precipitation is 450 mm, and most rainfall occurs in summer, whereas little rainfall and high evaporation are observed in the other three seasons. The ≥10 °C accumulated active temperature is about 2, 600 °C, annual sunshine duration is 2, 662 h, and the frost-free period is approximately 120 d. The soil type is Loess light brown soil. The initial soil properties in 2018 (0–20 cm depth) were as follows: organic matter: 12.5 g kg^−1^, total N (TN): 0.97 g kg^−1^, total phosphorus (P): 0.66 g/kg^−1^, total potassium (K): 19.24 g kg^−1^, available N: 69.88 mg kg^−1^, available P: 7.0 mg kg^−1^, available K: 91.0mg kg^−1^, pH: 8.25. The area depends mainly on natural precipitation for agricultural cultivation. The amount of precipitation during the growth period is shown in **Supplementary Table 1**.

### 2.2 Experimental design and field management

The experiment was carried out based on a split-plot design for two consecutive years from 2018 to 2019 with N application rate as the main factor and planting density as the second factor, with three replicates for each treatment. Four N application rates were applied, including 0 kg ha^−1^ (N0), 120 kg ha^−1^ (N120), 180 kg ha^−1^ (N180), and 240 kg ha^−1^ (N240). Two planting densities, including 45, 000 plants ha^−1^ (D4.5) and 60, 000 plants ha^−1^ (D6.0), were applied. The tested hybrid was Jinxiannuo 41, which is planted widely in Shanxi province. The size of each plot was 24 m^2^ (4.8 m × 5 m) with 0.6-m row spacing. N fertilizer (urea) and 120 kg P_2_O_5_ ha^−1^ were applied before sowing as base fertilizer. Other agronomic practices were consistent with local standard field management approaches for fresh *waxy* maize production. The experimental field was not subjected to irrigation. The dates of sowing and harvesting are listed in **Supplementary Table 2**.

### 2.3 Plant sampling and measurements

#### 2.3.1 Stalk agronomic characteristics and leaf area of three ear leaves

The plant height, ear height, and diameter of 10 randomly selected plants were measured at the silking stage in the middle rows of each plot. Plant height (from the ground to the top of the tassel) and ear height (from the ground to the node bearing the ear) were measured using a ruler. The diameters of the 3rd basal internode were also measured with a digital vernier caliper (0.01 mm). Accounting for 45% of the total photosynthetic leaf area of maize, the three ear leaves, with their longest functional duration and higher chlorophyll content, serve as the core functional leaves for yield formation (Fan et al., 2023; Wei et al., 2016; Xu et al., 2018). Five plants were sampled at the tasseling stage and the leaf area of three ear leaves was calculated as the length of three ear leaves × width of three ear leaves × 0.75. LAI of three ear leaves refers to the land area occupied by the LA × the number of plants per unit area.


LAI of three ear leaves = (Leaf area of three ear leaves× Plant population)/Plot area

#### 2.3.2 Dry matter and nitrogen accumulation

Maize plants were selected randomly from each plot at the R1 and late R3 stage (fresh waxy maize harvested). The aboveground parts of fresh waxy maize were harvested and divided into two parts at the R1 stage: leaves (bract), stems, and four parts at the R3 stage: leaves (bract), stems, grain, and cobs. The fresh plants were desiccated for 30 min at 105 °C, and then oven-dried at 80 °C to constant weight for the determination of DM accumulation. Dry samples were ground to a powder and passed through a 100-mesh sieve for use in determination of TN using an element analyzer. The post-silking DM (Post DM) accumulation and N uptake were calculated at the whole-plant level, and the DM and N allocations to different organs were quantified as the proportions of DM and N in different plant organs to total DM and total N of the whole plant. The post DM accumulation, N uptake, and DM and N proportions were calculated as follows:


$$Post\;DM\;per\;plant\;\left(g\;plant^{-1}\right)\;=\;whole\;plant\;DM_{R3}-\;whole\;plant\;DM_{R1}$$

$$Population\;DM\;\left(kg\;ha^{-1}\right)\;=\;DM\;per\;plant\;\times \;planting\;density$$

$$Post\;DM\;of\;population\;\left(kg\;ha^{-1}\right)\;=\;Post\;DM\;per\;plant\;\times \;planting\;density$$

$$Post\;N\;per\;plant\;\left(g\;plant^{-1}\right)\;=\;whole\;plant\;NR_{3}-\;whole\;plant\;NR_{1}$$

$$DM\;proportion\;\left(\%\right)\;=\;DM\;of\;specific\;organ/whole\;plant\;DM\;\times \;100\%$$

$$N\;proportion\;\left(\%\right)\;=\;N\;of\;specific\;organ/whole\;plant\;N\;\times \;100\%$$

#### 2.3.3 Fresh waxy maize yield

At the late R3 stage, the fresh ears with the bracts of the middle rows (2 rows × 5 m) were collected continuously from each plot to determine fresh ear yield. Ten representative ears were sampled to measure the weight per ear with bracts. The ear length was measured using a ruler. The ear diameter was measured with a digital vernier caliper (0.01 mm).


$$Nitrogen\;partialfactorproductivity\;\left(NPFP\right)\;=\;fresh\;ear\;yield/nitrogen\;application\;rate$$

### 2.4 Statistical analysis

MS Excel 2010 (Microsoft Corp., Redmond, WA, USA) was used for basic data analyses. All the statistical analyses were conducted in IBM SPSS Statistics 25 (IBM Corp., Armonk, NY, USA). Means were tested using the least significant difference tests at the P< 0.05 level (LSD 0.05). The figures were drawn using OriginPro 2016 (OriginLab Corp., Northampton, MA, USA).

## 3 Results

### 3.1 Stalk agronomic characteristics and leaf area index of three ear leaves

N application rate and planting density influenced stalk agronomic characteristics of fresh waxy maize significantly (**Table 1**). All stalk agronomic characteristics increased with an increase in N application rate and were the highest under the N120 and N180 treatments, and then decreased slightly. Average plant height, ear height, stem diameter, and LAI of three ear leaves under the two planting densities and the N120–N240 treatments increased significantly by 10.3%–12.2%, 14.6%–18.8%, 10.6%–43.4%, and 24.3%–29.7%, respectively, when compared with those in the N0 treatment. In contrast, increasing planting density had no significant effect on plant or ear height, but induced significant reduction in stem diameter. Based on the two-year average, stem diameter in the D6.0 treatment was significantly lower than that in the D4.0 treatment, by 5.5%–13.0%, across the four N application treatments. The most pronounced effect of the high density treatment (D6.0) was a substantial increase in LAI of the three ear leaves, which were on average 31.8% higher than those in the D4.5 treatment across all N application rates. The results demonstrate that optimal N application (N120–N180) promoted stalk growth, while higher planting density improved canopy LAI at the cost of reduced stem thickness.

**Table 1:** Effects of nitrogen application rate and planting density on stalk agronomic characteristic and LAI of fresh waxy maize.

Year	N application rate	Planting density	Plant height (cm)		Stem diameter (mm)		Ear height (cm)		Leaf area index of three ear leaves	
2018	N0	D4.5	210.4	d	24.0	b	79.3	c	0.613	f
		D6.0	199.2	e	20.7	c	74.9	d	0.980	c
	N120	D4.5	215.9	c	25.4	a	82.1	c	0.831	e
		D6.0	224.1	a	24.1	b	91.8	a	1.225	a
	N180	D4.5	220.8	ab	25.7	a	82.4	c	0.960	cd
		D6.0	218.3	bc	24.1	b	86.6	b	1.169	b
	N240	D4.5	219.3	bc	25.7	a	86.3	b	0.925	d
		D6.0	215.9	c	23.6	b	87.9	b	1.161	b
2019	N0	D4.5	188.6	c	21.8	c	76.0	d	0.888	d
		D6.0	177.4	d	19.1	d	74.5	d	0.824	e
	N120	D4.5	212.4	ab	25.3	a	94.0	ab	0.851	de
		D6.0	217.9	a	23.7	ab	94.3	a	1.204	b
	N180	D4.5	207.3	b	24.6	a	87.3	c	0.886	d
		D6.0	212.0	ab	22.1	bc	92.9	abc	1.247	a
	N240	D4.5	210.3	b	25.3	a	89.6	abc	0.932	c
		D6.0	210.4	b	23.7	ab	87.8	bc	1.269	a
Source of variation										
Year (Y)			**		**		**		**	
N application rate (N)			**		**		**		**	
Planting density (D)			ns		**		ns		**	
Y × N			**		**		**		**	
Y × D			ns		ns		ns		**	
N × D			**		ns		**		**	
Y × N × D			ns		ns		ns		**	

N0, N120, N180, and N240 represent N application rates of 0, 120, 180, and 240 kg ha^−1^, respectively; D4.5 and D6.0 represent planting densities of 45, 000 and 60, 000 plants ha^−1^, respectively. Different lowercase letters after data in the same column indicate significant differences among treatments in the same year at the 0.05 level. *, **, and ns represent differences at significant levels of 0.05, 0.01, and no significant difference, respectively.

### 3.2 Fresh waxy maize yield and nitrogen partial factor productivity

**Figure 1:**
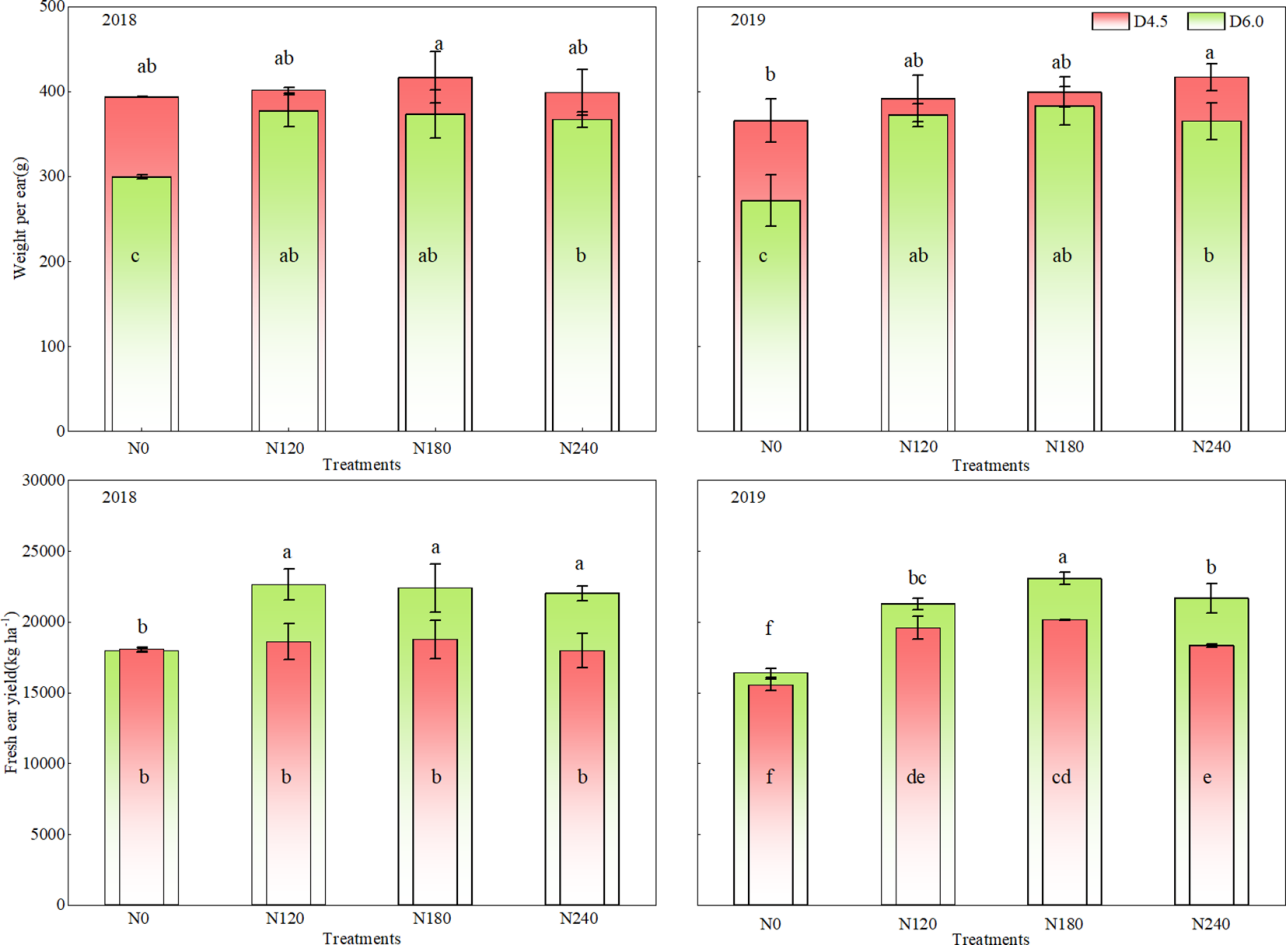
Effects of nitrogen (N) application rate and planting density on ear yield and weight per ear of fresh waxy maize. N0, N120, N180, and N240 represent N application rates of 0, 120, 180, and 240 kg ha^−1^, respectively; D4.5 and D6.0 represent plant densities of 45, 000 and 60, 000 plants ha^−1^, respectively. Different lowercase letters on or in the bar indicate significant differences among treatments in the same year at *P*< 0.05.

**Figure 2:**
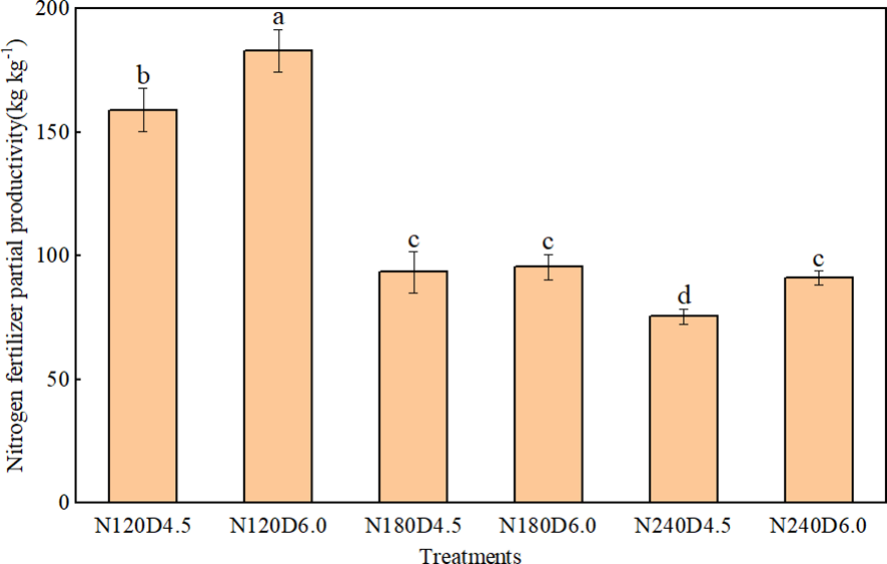
Effect of planting density and nitrogen rate on nitrogen fertilizer partial productivity of fresh waxy maize (2018–2019 average). N0, N120, N180, and N240 represent N application rates of 0, 120, 180, and 240 kg ha^−1^, respectively; D4.5 and D6.0 represent plant densities of 45, 000 and 60, 000 plants ha^−1^, respectively. Different lowercase letters indicate significant differences among different treatments (*P*< 0.05).

Both N application rate and planting density affected fresh ear yield, weight per ear, NPFP of waxy maize significantly (**Table 2**). Increasing planting density consistently enhanced fresh ear yield but reduced weight per ear (**Figure 1**). In the two-year study period, fresh ear yield under the D6.0 treatment increased significantly, by 4.6%–21.4%, whereas weight per ear decreased by 5.5%–24.8%, across the four N application rates, when compared with those under the D4.5 treatment. Fresh ear yield increased first and then decreased with an increase in N application rate, with maximum yields under the N120 and N180 treatments in 2018 and 2019, respectively. NPFP exhibited an increasing trend with an increase in planting density and a decrease in N rate (**Figure 2**). In addition, increasing planting density enhanced NPFP by 2.2%–20.5%. Compared with the N240 rate, the N180 rate increased NPFP by 23.6% under the D4.5 treatment and by 4.9% under the D6.0 treatment. The N180 and D6.0 combination was the optimal treatment for higher yield and NPFP, with no significant reduction in weight per ear, when compared with that under the N240 rate.

**Table 2:** Effects of nitrogen application and planting density on fresh ear yield and nitrogen fertilizer partial productivity (NPFP).

Factor	Year (Y)	N application rate (N)	Planting density (D)	Y × N	Y × D	N × D	Y × N × D
Fresh ear yield	ns	**	**	*	ns	**	ns
Weight per ear	**	**	**	*	ns	*	ns
Ear length	**	**	ns	**	ns	ns	**
Ear diameter	*	**	**	*	*	ns	ns
NPFP	ns	**	**	ns	ns	**	ns

*, ** and ns represent significant difference levels of 0.05, 0.01, and no significant difference, respectively.

N application rate significantly influenced ear length and diameter, whereas planting density significantly influenced only ear diameter (**Table 2**). Based on the two-year average, ear diameter in the D6.0 treatment was lower than that in the D4.0 treatment by 0.12 cm. Average ear length, ear diameter under the two planting densities and the N120–N240 treatments increased significantly by 0.21cm-1.05cm, 0.12 cm -0.24cm, respectively, when compared with those in the N0 treatment. Ear length and diameter increased first and then decreased with an increase in N application rate, with maximum values under the N180 treatments, respectively (**Table 3**). Although increasing planting density tended to reduce ear length and ear diameter in fresh waxy maize, an appropriate nitrogen application rate (N180) can effectively enhance both these traits.

**Table 3:** Effects of nitrogen application rate and planting density on ear length and diameter of fresh waxy maize.

Year	N application rate	Planting density	Ear length(cm)		Ear diameter(cm)	
2018	N_0_	D_4.5_	18.38	cd	4.88	bcd
		D_6.0_	20.04	ab	4.62	d
	N_120_	D_4.5_	19.75	ab	4.85	bcd
		D_6.0_	19.18	bc	4.80	bcd
	N_180_	D_4.5_	20.27	a	5.16	a
		D_6.0_	20.03	ab	4.95	ab
	N_240_	D_4.5_	19.10	bc	4.90	bc
		D_6.0_	17.92	d	4.65	cd
2019	N_0_	D_4.5_	19.75	bc	4.58	c
		D_6.0_	19.13	c	4.53	c
	N_120_	D_4.5_	20.25	ab	4.66	bc
		D_6.0_	19.88	abc	4.80	ab
	N_180_	D_4.5_	20.75	a	4.82	a
		D_6.0_	20.43	ab	4.65	bc
	N_240_	D_4.5_	20.38	ab	4.87	a
		D_6.0_	20.75	a	4.75	ab

N0, N120, N180, and N240 represent N application rates of 0, 120, 180, and 240 kg ha^−1^, respectively; D4.5 and D6.0 represent planting densities of 45, 000 and 60, 000 plants ha^−1^, respectively. Different lowercase letters after data in the same column indicate significant differences among treatments in the same year at the 0.05 level.

### 3.3 Dry matter accumulation

**Figure 3:**
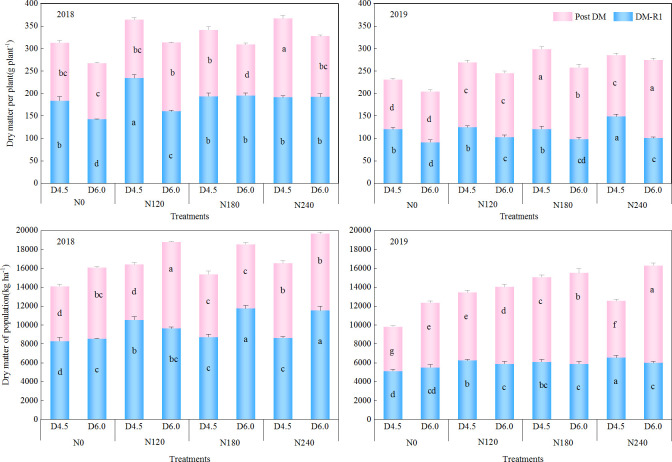
Effects of nitrogen application rate and planting density on dry matter accumulation at the R1 and post-silking stages. N0, N120, N180, and N240 represent N application rates of 0, 120, 180, and 240 kg ha^−1^, respectively; D4.5 and D6.0 represent plant densities of 45, 000 and 60, 000 plants ha^−1^, respectively. Different lowercase letters on the bar indicate significant differences among treatments in the same year at *P*< 0.05.

DM accumulation was influenced significantly by N application and planting density, revealing distinct patterns at the individual and population levels (**Figure 3**). Increasing planting density decreased DM accumulation per plant; conversely, it increased DM accumulation at the population level across all N application rates. In the two-year study period, DM accumulation per plant under the D6.0 treatment across the four N application rates decreased by 6.7%–26.7% at the R1 stage and by 7.8%–13.2% at the late R3 stage, whereas DM accumulation in the population increased by 4.8%–9.0% at the R1 stage and by 10.0%–23.7% at the late R3 stage, when compared with those under the D4.5 treatment. N application augmented both per-plant and population level DM accumulation. Moreover, DM accumulation per plant under the N120–N240 treatments was increased by 13.1%–17.8% at the R1 stage and by 17.4%–23.5% at the late R3 stage, whereas DM accumulation in the population increased by 17.7%–19.2% at the R1 stage and by 19.8%–24.3% at the late R3 stage, when compared with those under the N0 treatment. The results indicate that increasing planting density required a proportionate increase in N application to enhance population DM accumulation, as the basis for yield formation.

### 3.4 Nitrogen accumulation

**Figure 4:**
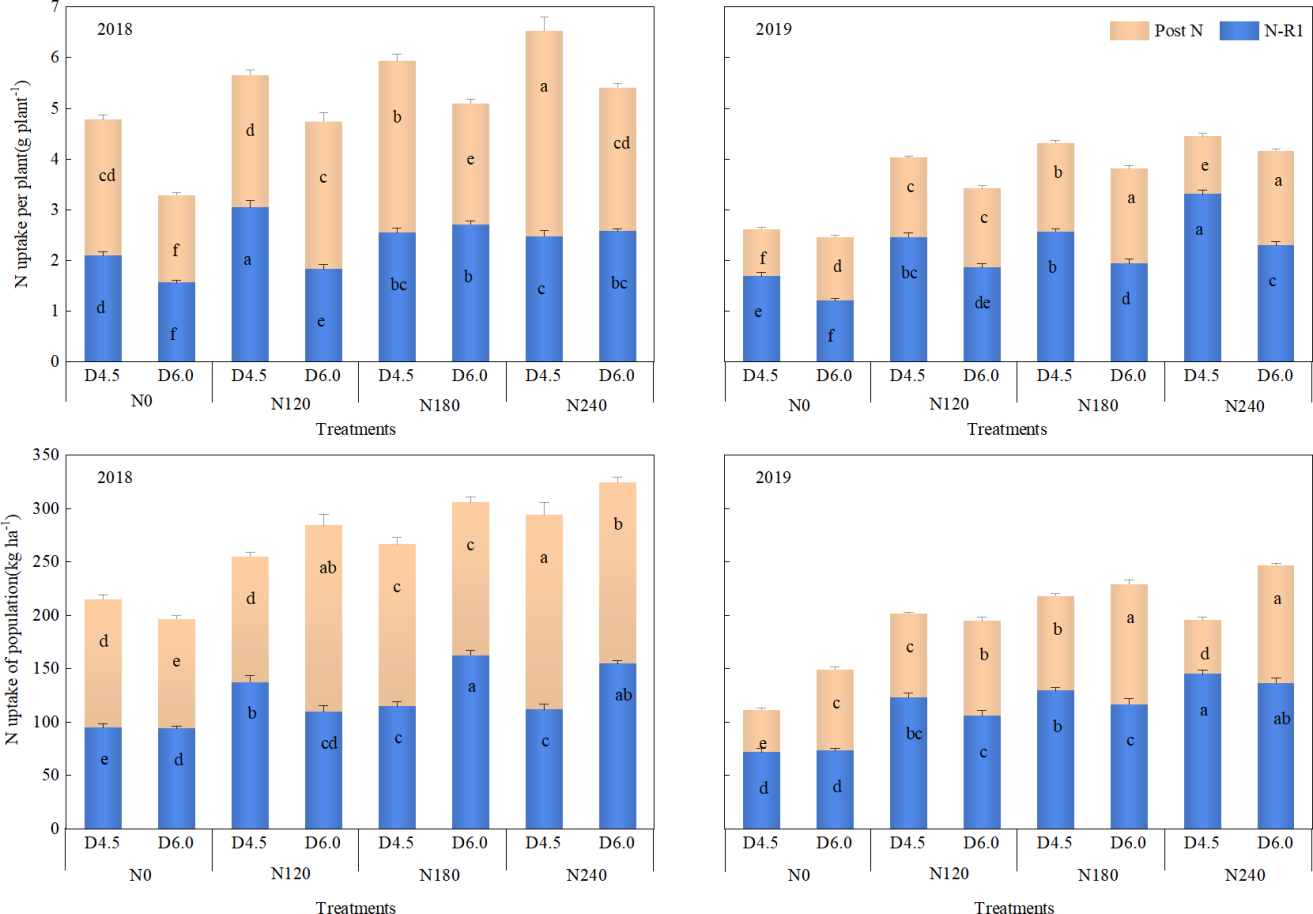
Effects of nitrogen application rate and planting density on nitrogen accumulation at the R1 and post silking stages. N0, N120, N180, and N240 represent application rates of 0, 120, 180, and 240 kg ha^−1^, respectively; D4.5 and D6.0 represent plant densities of 45, 000 and 60, 000 plants ha^−1^, respectively. Different lowercase letters on the bar indicate significant differences among treatments in the same year at *P*< 0.05.

N application and planting density affected waxy maize N uptake significantly (**Figure 4**). Increasing planting density decreased N uptake per plant, but increased N uptake at the population level. In the two year study, the D6.0 treatment decreased N uptake per plant by 9.4%–33.3% at R1 stage and 12.9%–22.3% at the late R3 stage, and it increased population N uptake by 0.5%–14.1% at the R1 stage and by 4.9%–16.8% at the late R3 stage, when compared with those in the D4.5 treatment. Conversely, increasing N application rate enhanced both per-plant and population level N uptake at the two growth stages. The findings demonstrate that high-density planting requires adequate N supply to sustain per-plant and population-level N uptake for achievement of higher yield.

### 3.5 Proportions of dry matter and nitrogen content in different organs

**Figure 5:**
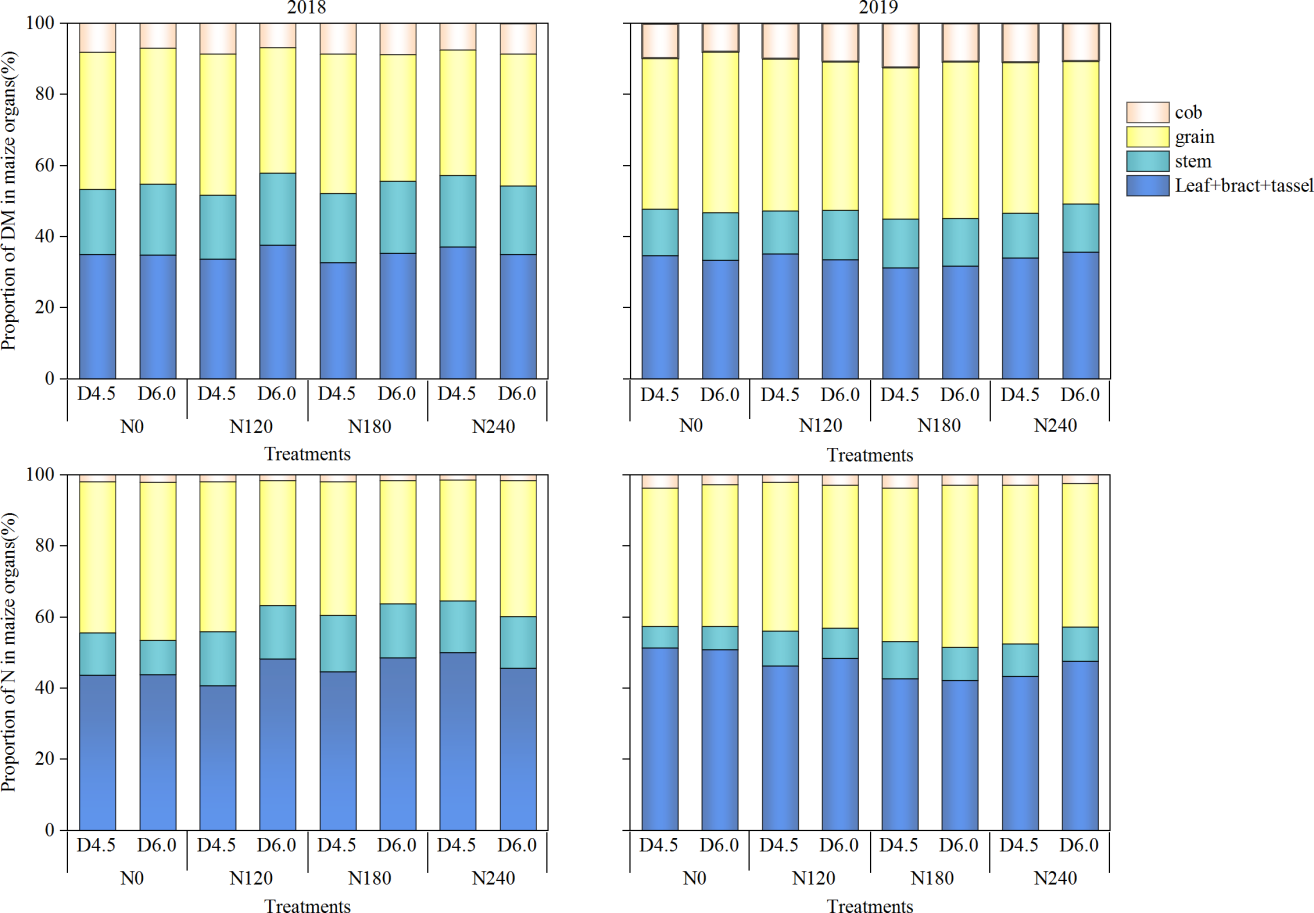
Proportions of dry matter (DM) and nitrogen (N) in different plant organs out of whole plant under different N application rates and planting densities at the late R3 stage. N0, N120, N180, and N240 represent N application rates of 0, 120, 180, and 240 kg ha^−1^, respectively; D4.5 and D6.0 represent plant densities of 45, 000 and 60, 000 plants ha^−1^, respectively. Different lowercase letters on the bar indicate significant differences among treatments in the same year at *P*< 0.05.

N application rate and planting density influenced DM and N allocations to maize ears (cob + grain) (**Figure 5**). The proportion of DM in ears was the maximum under the N180 treatment but generally decreased with an increase in planting density. Compared with the D4.5 treatment, the D6.0 treatment reduced the proportion of DM in ear (cob + grain) by 0.37%–6.56% under the N0, N120 and N180 treatments at the late R3 stage, and it decreased the proportion of N in ear (cob + grain) by 0.25%–9.34%% under the N120, N180, and N240 treatments, respectively.

### 3.6 Correlation between fresh waxy maize yield and indictors

A correlation analysis was carried out to analyze the relationship between fresh ear yield and DM accumulation and distribution, and between fresh ear yield and N accumulation and distribution. Fresh ear yield was most positively correlated with population-level indicators, including LAI, Post DM accumulation of the population, DM accumulation of the population at the R3 stage, and N accumulation of the population (**Table 4**). In contrast, fresh ear yield demonstrated weak or non-significant correlations with per-plant accumulation indicators and DM and N allocation to different organs. The results indicate that under high-density planting conditions, LAI and efficient accumulation at the population levels influence fresh ear yield, rather than individual plant performance.

**Table 4:** Correlation analysis of fresh waxy maize yield and indictors.

Indictors		EY
population-level indicators	LAI	0.799**
	DM-R1(kg ha^-1^)	0.315
	Post DM(kg ha^-1^)	0.736**
	DM-late R3(kg ha^-1^)	0.713**
	N-R1(kg ha^-1^)	0.624**
	Post N(kg ha^-1^)	0.472
	N- late R3(kg ha^-1^)	0.641**
per-plant accumulation indicators	DM-R1(g plant^-1^)	-0.023
	Post DM((g plant^-1^)	0.409
	DM- late R3((g plant^-1^)	0.173
	N-R1((g plant^-1^)	0.178
	Post N((g plant^-1^)	0.208
	N- late R3(g/(g plant^-1^)	0.242
DM and N allocation to different organs	DM(Leaf+bract+tassel)	0.048
	DM(stem)	0.147
	DM(grain)	-0.282
	DM(cob)	0.210
	N(Leaf+bract+tassel)	-0.184
	N(stem)	0.369
	N(grain)	-0.116
	N(cob)	-0.257

Correlation coefficients are determined by Pearson’s correlation. Data are the means of the two years;.

EY, fresh ear yield. LAI, leaf area index. DM-R1(kg ha^-1^), population DM accumulation at R1 stage. Post DM(kg ha^-1^), population DM accumulation post-silking. DM- late R3(kg ha^-1^), population DM accumulation at late R3 stage. N-R1(kg ha^-1^), population N uptake at R1 stage. N-late R3(kg ha^-1^), population N uptake at late R3 stage. Post N(kg ha^-1^), population N uptake post-silking.

DM-R1(g plant^-1^), DM accumulation per plant at R1 stage. Post DM(g plant^-1^), DM accumulation per plant post-silking. DM- late R3(g plant^-1^), DM accumulation per plant at late R3 stage. N-R1(g plant^-1^), N uptake per plant at R1 stage. N- late R3(g plant^-1^), N uptake per plant at late R3 stage. Post N(g plant^-1^), N uptake per plant post-silking.

DM (Leaf + bract + tassel), fraction of DM in leaves, bracts, and tassels. DM (stem), fraction of DM in stems. DM (grain), fraction of DM in grains. DM (cob), fraction of DM in cobs. N (Leaf + bract + tassel), fraction of N in leaves, bracts, and tassels. N (stem), fraction of N in stems. N (grain), fraction of N in grains. N (cob), fraction of N in cobs.

* and ** represent significant differences at *P*< 0.05 and *P*< 0.01, respectively.

## 4 Discussion

### 4.1 Effects of nitrogen application rate and planting density on ear yield and nitrogen partial factor productivity

Agronomic practices, particularly planting density and fertilization, are key factors that influence the yield and quality of maize (Lu et al., 2024; Shi et al., 2016; Wu et al., 2023). Studies on N fertilization and plant density on maize yield have been widely reported, yet the optimal N application rates and planting density for maize varied by different ecological regions and maize cultivars (Hou et al., 2023; Lei et al., 2024; Zhai et al., 2022). However, compared to grain maize, limited research has focused on planting density and N fertilization in per-plant or population production of fresh waxy maize, which is harvested at the late R3 stage (Gao et al., 2022). Fresh waxy maize yield is a function of both ears per unit area and fresh weight per ear. Therefore, it is imperative to determine the appropriate N rates and planting densities for achieving comparable weight per ear to low-density stands and maximizing population-level fresh ear yield.

The synergistic interaction between dense planting and an appropriate N rate can fully exploit the population yield potential of maize, thereby facilitating achievement of a balanced nutrient supply-demand relationship and the dual objectives of high yield and high efficiency (Cheng et al., 2020; Fan et al., 2024). Increasing planting density increased the extinction coefficient, solar radiation interception, and the utilization efficiency of light and nitrogen resources, thereby increasing crop yield (Lei et al., 2024). Additionally, high density improved soil microbial biomass and diversity, thus reconciling maize productivity and soil health (Hou et al., 2023). The present study indicated that three ear leaf LAI increased with rising planting densities and nitrogen rates (**Table 1**), which is consistent with previous findings (Li et al., 2026). The establishment of source organs (leaf quantity and area) is fundamental to yield formation. According to the results of the present study, increasing planting density (D6.0) reduced weight per ear but increased ear number per unit area, leading to a compensatory effect that raised fresh ear yield, which is consistent with previous findings (Xiao et al., 2012). This reveals a clear trade-off between population-level yield and individual ear weight (Yao et al., 2023). Regarding N application, the highest fresh ear yield was obtained under N120 in 2018 and under N180 in 2019, and increasing N rate did not increase fresh ear yield indefinitely (**Figure 1**). Our results further demonstrate that optimal N supply can mediate the trade-off between individual performance and population productivity. This aligns with previous findings that increasing N rate did not necessarily result in a proportional increase in maize yield (Duan et al., 2023; Hou et al., 2023), indicating that surplus N was not used effectively by maize plants (Xu et al., 2017). Compared with high N application rate, moderate nitrogen reduction could fully utilize the available nitrogen from the fertilizer applied and the soil, thereby reducing nitrogen loss, significantly improve the N fertilizer apparent use efficiency (Lei et al., 2024). Excessive nitrogen application (400 kg N ha^-1^) disrupted the stability of soil microbial communities, inhibited certain key functional microorganisms (e.g., ammonia-oxidizing bacteria), and led to an imbalance in microbial community structure, resulting in no significant increase in yield compared with the 200 kg N ha^−1^ treatment (Hou et al., 2023). Notably, the highest yield was observed under the N180 and D6.0 combination, and negligible disparities in weight per ear were observed between the D6.0 and D4.5 treatments (**Figure 1**). Therefore, an appropriate N rate and an increase in planting density would achieve high fresh ear yield, with no significant reduction in weight per ear.

### 4.2 Effects of nitrogen application rate and planting density on dry matter and nitrogen accumulation and distribution

Crop production is essentially a population process rather than an simple individual plant performance. Appropriate agronomic practices can optimize population structure to increase DM and nutrient accumulation and allocation to sink organs, thereby improving crop yield (Chen et al., 2022; Li et al., 2020; Yang et al., 2025).In the present study, compared with the D4.5 treatment, the D6.0 treatment increased population DM by 4.8%–9.0% at the R1 stage and10.1%–23.7% at the late R3 stage (**Figure 3**) and population N uptake by 6.7%–26.7% at the R1 stage and 7.8%–13.2% at the late R3 stage (**Figure 4**). This suggests that increasing planting density leads to a greater increase in post-anthesis dry matter accumulation than in pre-anthesis accumulation. The elevated post-anthesis dry matter accumulation, in turn, reduces the excessive remobilization of pre-anthesis assimilates, which accelerates leaf senescence, thereby sustaining a robust source supply during the critical grain-filling period, and consequently establishing a foundation for yield formation (Li et al., 2026; Liu et al., 2020).

Notably, although high planting density optimizes population-level productivity, it intensifies competition for water, nutrients, and light among adjacent individual plants. In previous studies, increased planting density decreased chlorophyll content and photosynthetic rate, leading to decreases in single-plant biomass accumulation (Lan et al., 2024; Yan et al., 2024). By contrast, N application serves as a critical regulatory measure to alleviate such adverse density-induced physiological limitations (Guo et al., 2021; Li et al., 2026). In agree with these previous studies, our results also confirmed that as N application rates increase, both individual plant and population-level DM accumulation and N uptake exhibited upward trends (**Figures 3**, **4**). Therefore, these findings highlight that synchronizing N application with planting density could enhance N and DM accumulation per plant, thereby improving population DM accumulation and N uptake (Lu et al., 2024; Yao et al., 2023).

Beyond population-level accumulation, the rational allocation of N to the ear is also fundamental for high crop yield (Duan et al., 2023). In the present study, as planting density increased, the proportion of N allocation to ear decreased, while the proportion of N allocated to vegetative organs (e.g., leaves and stems) increased. This shift enhanced the photosynthetic capacity of the population, thereby realizing the advantages of dense planting (Yao et al., 2023). However, on the other hand, high-density conditions intensify competition among adjacent individuals (Zhai et al., 2018), leading to restricted per-plant N accumulation and reduced N supply available for remobilization to sink organs during the post-anthesis period, thereby limiting dry matter accumulation. Therefore, adequate N application to enhance plant N accumulation before the onset of linear grain filling can extend grain-filling duration and increase kernel N accumulation rate, thereby promoting dry matter accumulation (Olmedo Pico and Vyn, 2021). Our findings suggested that appropriate N application increases the distribution of DM and N to the ear (**Figure 5**). This demonstrates that optimizing N management is key to mitigating the negative impacts of high density on nutrient allocation to sink organs. Reducing N input while increasing planting density (N2D2) can simultaneously leverage the source advantages of a high-density population and ensure sufficient N allocation to the ear. Therefore, reducing nitrogen application combined with increasing planting density can maintain a balanced source-sink relationship, avoiding yield limitation caused by sink limitation and source surplus, as well as yield losses from inadequate post-anthesis source supply caused by excessive nitrogen remobilization (Li et al., 2026; Wei et al., 2018).

### 4.3 Correlation between fresh waxy maize yield and indictors

Although fresh waxy maize and grain maize differ in harvest timing (late R3 vs. R6), both undergo the same early grain-filling processes after silking. Specifically, during the lag phase, kernel development is characterized by endosperm cell division with negligible dry weight gain. After this phase, kernels enter the effective grain-filling phase, where they actively accumulate DM at a constant rate (Olmedo Pico and Vyn, 2021). Previous studies have indicated that dry matter accumulation constitutes the material basis for maize yield formation (Liu et al., 2023b), and grain yield exhibits a strong linear correlation with nitrogen uptake, assimilation, and distribution within the plant (Guo et al., 2022). The results of the present study showed that fresh ear yield was most positively correlated with population-level indicators, but had weak or non-significant correlations with per-plant accumulation indicators and the partitioning of DM and N to different organs (**Table 4**). Our findings reinforce the perspective that in high-density systems, yield is predominantly a function of population-level factors (Cheng et al., 2020; Guo et al., 2025). Therefore, increasing planting density is appropriately a crucial strategy for enhancing yield by boosting DM accumulation and N uptake at the population level (Fan et al., 2024; Li et al., 2022c). In particular, post-silking DM accumulation and pre-silking N accumulation played crucial roles in the yield formation in fresh waxy maize. Our results are consistent with the findings of previous studies, which have indicated that post-silking leaf photosynthesis could adequately supply the assimilates for grain DM (Fan et al., 2023), and approximately a half of grain N originates from pre-silking absorption via N remobilization from the vegetative organs during the post-silking stage (Li et al., 2022b). Appropriate N application and planting density increased DM accumulation and N uptake at the population level, especially post-silking DM accumulation and pre-silking N uptake, thereby increasing ear yield of fresh waxy maize.

The present study offers insights that could guide the optimization of N application and planting density for the production of fresh waxy maize, an economically important crop, in similar regions globally. However, this study was conducted based on two years of field measurements, and long-term experiments are still required to determine the optimal N–density combinations under diverse climatic conditions, especially varying precipitation patterns. Furthermore, the current study only analyzed phenotypic changes in DM and N accumulation and allocation, while the underlying physiological mechanisms regulating source−sink relationships were not fully elucidated. Therefore, future studies should further investigate key physiological processes, including photosynthetic performance, root N uptake activity, and N translocation dynamics during the critical pre−silking and post−silking stages. Such efforts will help clarify the physiological pathways by which N supply and planting density jointly modulate source−sink balance, thereby improving the mechanistic understanding of fresh waxy maize production and providing reliable support for region-specific cultivation management strategies.

## 5 Conclusion

Increasing planting density decreased DM and N uptake per plant, reduced the allocation of DM and N to ear, while increasing population-level DM accumulation and N uptake. An optimal N application rate of 180 kg N ha^−1^ enhanced both individual and population DM accumulation and distribution to the ear, as well as plant N uptake. The present study reveals that achieving high fresh ear yield in rain-fed waxy maize hinges on balancing the inherent trade-off between individual plant performance and population-level productivity. The combination of 180 kg N ha^−^¹ and 60, 000 plants ha^−^¹ achieves such a balance by offsetting density-induced individual penalties through optimized N supply. These findings provide a practical guidance for optimizing N application and planting density management to enhance DM accumulation and N uptake, as well as fresh waxy maize yield in the study area or in other regions with similar climatic conditions.

## Data Availability

The original contributions presented in the study are included in the article/**Supplementary Material**. Further inquiries can be directed to the corresponding author.
