# Supplementary material for: Balancing individual trade-offs for population gain: optimizing nitrogen and density secures fresh ear yield in rain-fed waxy maize
Source: Front Plant Sci. 2026 May 22;17:1831472. doi: 10.3389/fpls.2026.1831472 (PMC13236957; doi:10.3389/fpls.2026.1831472)
Supplement: Supplementary file 1 [file Table1.docx]

**Table S1.** Precipitation in the experimental fields during the fresh waxy maize growth period in 2018 and 2019

| **Year** | **Precipitation (mm)** | | | | |
| --- | --- | --- | --- | --- | --- |
|  | **May** | **June** | **July** | **August** | **Total** |
| 2018 | 46.2 | 46.5 | 132.0 | 75.7 | 300.4 |
| 2019 | 3.6 | 90.5 | 79.1 | 109.2 | 282.4 |
| Mean precipitation | 32.8 | 59.4 | 101.6 | 89.7 | 283.5 |

**Table S2.** Dates of sowing and harvesting

| **Year** | **Sowing date (Month-Date)** | **Harvesting date (Month-Date)** |
| --- | --- | --- |
| 2018 | 05-08 | 08-10 |
| 2019 | 05-09 | 08-16 |
